# Reprogramming the metabolism of an acetogenic bacterium to homoformatogenesis

**DOI:** 10.1038/s41396-023-01411-2

**Published:** 2023-04-15

**Authors:** Jimyung Moon, Anja Schubert, Lara M. Waschinger, Volker Müller

**Affiliations:** Molecular Microbiology & Bioenergetics, Institute of Molecular Biosciences, Johann Wolfgang Goethe University, Max-von-Laue Str. 9, D-60438 Frankfurt, Germany

**Keywords:** Microbiology, Metabolism, Mutation, Environmental microbiology, Microbial ecology

## Abstract

Methyl groups are abundant in anoxic environments and their utilization as carbon and energy sources by microorganisms involves oxidation of the methyl groups to CO_2_, followed by transfer of the electrons to an acceptor. In acetogenic bacteria, the electron acceptor is CO_2_ that is reduced to enzyme bound carbon monoxide, the precursor of the carboxyl group in acetate. Here, we describe the generation of a mutant of the acetogen *Acetobacterium woodii* in which the last step in methyl group oxidation, formate oxidation to CO_2_ catalyzed by the HDCR enzyme, has been genetically deleted. The mutant grew on glycine betaine as methyl group donor, and in contrast to the wild type, formed formate alongside acetate, in a 1:2 ratio, demonstrating that methyl group oxidation stopped at the level of formate and reduced electron carriers were reoxidized by CO_2_ reduction to acetate. In the presence of the alternative electron acceptor caffeate, CO_2_ was no longer reduced to acetate, formate was the only product and all the carbon went to formate. Apparently, acetogenesis was not required to sustain formatogenic growth. This is the first demonstration of a genetic reprogramming of an acetogen into a formatogen that grows by homoformatogenesis from methyl groups. Formate production from methyl groups is not only of biotechnological interest but also for the mechanism of electron transfer in syntrophic interactions in anoxic environments.

## Introduction

Methyl groups serve as carbon and energy sources for microbial growth in anoxic environments; they are oxidized to CO_2_ with concomitant reduction of electron carriers such as NAD^+^, NADP^+^ or ferredoxin. Reoxidation of these electron carriers is coupled to the reduction of internal or external electron acceptors. In methanogens, methyl groups also serve as electron acceptors and are reduced to methane [1, 2], in sulfate reducers external sulfate is reduced to sulfide [3–5] or in acetogens, CO_2_ is reduced to enzyme bound CO, followed by condensation with a methyl group to yield acetate [6–8]. In the latter group, the methyl group is transferred by a methyltransferase system to the central pathway, the Wood-Ljungdahl pathway (WLP), by binding to tetrahydrofolate (THF), yielding methyl-THF [9, 10]. One enzyme of the methyltransferase system, methyltransferase I (MTI), is substrate specific and the importance of methyl groups for the nutrition of acetogens can be seen from the great number of different MTI enzymes [6, 7, 11–14]: in the acetogen *Acetobacterium woodii*, 23 different MTI enzymes are encoded [8, 15] and for only two is the natural substrate known (methanol and glycine betaine) [6, 7].

**Fig. 1:**
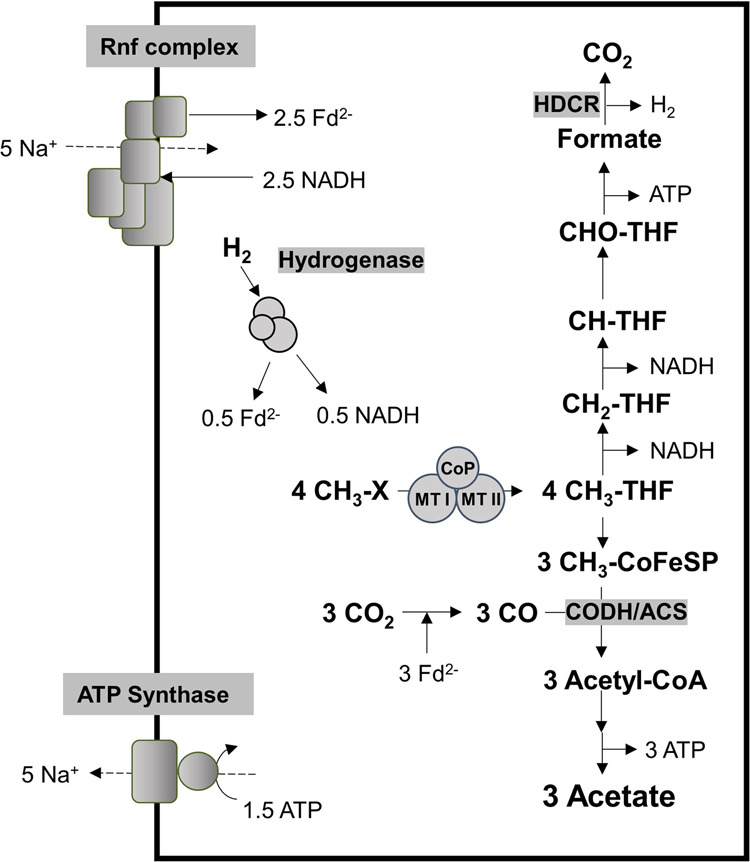
Biochemistry and bioenergetics of methylotrophic acetogenesis in *A. woodii*. CH_3_-X methyl groups, Fd ferredoxin, THF tetrahydrofolate, CODH/ACS CO dehydrogenase/acetyl coenzyme A synthase, CoFeSP corrinoid iron-sulfur protein, MTI methyltransferase I, MTII methyltransferase II, CoP corrinoid protein. The stoichiometry of the ATP synthase is 3.3 Na^+^/ATP [67] and for the Rnf complex a stoichiometry of 2 Na^+^/2 e^−^ is assumed.

Methyl-THF is then oxidized via methylene- and methenyl-THF to formate [6–8], which is further oxidized in *A. woodii* by the filamentous hydrogen-dependent carbon dioxide reductase (HDCR) [16–18] to carbon dioxide and molecular hydrogen (Fig. 1). NADH produced in the methylene-THF reductase and methylene-THF dehydrogenase reactions [19, 20] is reoxidized by reducing carbon dioxide to enzyme-bound carbon monoxide which is then condensed on the enzyme CO dehydrogenase/acetyl-CoA synthase (CODH/ACS) with another methyl group originating from methyl-THF to yield acetyl-CoA that is further converted to acetate. The CODH reaction specifically requires reduced ferredoxin as reductant [21, 22] but reduction of ferredoxin with NADH or molecular hydrogen is highly endergonic. This thermodynamic barrier is overcome by the membrane-integral Rnf complex that uses the transmembrane electrochemical Na^+^ gradient to drive this endergonic reaction [23] or by the soluble electron bifurcating hydrogenase that reduces ferredoxin and NAD^+^ in equimolar amounts with hydrogen as reductant [24]. In sum, acetate is synthesized from methyl group and carbon dioxide according to:

1 $$4\;{{{{{{{\mathrm{CH}}}}}}}}_3{{{{{{{\mathrm{ - X}}}}}}}} + 2\;{{{{{{{\mathrm{CO}}}}}}}}_2 \to 3\;{{{{{{{\mathrm{CH}}}}}}}}_3{{{{{{{\mathrm{COOH}}}}}}}} + {{{{{{{\mathrm{X}}}}}}}} + 2.5\;{{{{{{{\mathrm{ATP}}}}}}}}$$

This metabolism yields 0.63 mol of ATP per mol of methyl group.

Acetogenesis by disproportionation of methyl groups to acetate and CO_2_ is observed in pure cultures in the presence of CO_2_ in the gas phase. However, when methanol was fed in combination with other electron donors like H_2_ or CO, the fate of the methyl group changed: it was no longer oxidized but exclusively converted to acetate [25]. Moreover, in the presence of the alternative electron acceptor caffeate, methanol was oxidized only and caffeate was reduced, acetate was no longer produced [25, 26]. These different pathways for the conversion of methyl-group containing substrates enable acetogens to adapt to various ecological niches and to syntrophic communities [8].

In syntrophic communities, there is another way for the reoxidation of reduced electron carriers. These communities are characterized by interspecies electron transfer and the electron is transferred from one cell to the other as molecular hydrogen or as formate [27]. In *A. woodii* and other acetogens that have the HDCR enzyme, the oxidative and reductive parts of metabolism are connected by a hydrogen cycle [28]. Genetic deletion of the electron bifurcating hydrogenase led to a loss of growth on fructose and further experiments clearly revealed the presence of a hydrogen cycle in *A. woodii* [28]. Of course, hydrogen can escape from the cells into the environment and is scavenged by others, for example serving as reductant for CO_2_ reduction by methanogens. This “oxidation only mode” was observed during growth on fructose but also during growth on methanol in coculture of *A. woodii* with a H_2_-consuming methanogen that removed the hydrogen thereby enabling this lifestyle energetically by removing the end product [29, 30]. Formate and molecular hydrogen are in equilibrium in most ecosystems [27] and thus, formate is regarded as soluble electron carrier between species. However, formatogenesis from methanol or methyl groups has not been observed in strict anaerobes such as methanogens, sulfate reducers or acetogens. The latter are known to produce formate from H_2_ + CO_2_ transiently, indicating the capacity to export and import formate [31–33]. This prompted us to genetically engineer a formate producing pathway in the model acetogen *A. woodii*. Indeed, by deleting the *hdcr* gene cluster we turned *A. woodii* into a formate producer that grew non-acetogenically on methyl groups.

## Materials and methods

### Cultivation of *A. woodii*

*A. woodii* DSM1030 (wild type), ∆*hydBA*, ∆*hdcr* and ∆*hydBA/hdcr* were routinely cultivated under anoxic conditions at 30 °C in carbonate buffered complex medium as described previously [34]. Since all the mutants are derived from the ∆*pyrE* strain, 50 mg/l uracil was added to the medium [35]. As carbon and energy source, 50 mM glycine betaine was used. Growth was determined by measuring the optical density at 600 nm (OD_600_).

### Genetic modifications

Generation and characterization of the ∆*hydBA* mutant was described before [28]. To delete the *hdcr* operon (*fdhF1*, *hycB1*, *fdhF2*, *hycB2*, *fdhD*, *hycB3*, *hydA2*; Awo_c08190 – Awo_c08260), the plasmid pMTL_AW_KO_HDCR was generated in *E. coli* HB101 (Promega, Madison, WI, USA) and transformed into *A. woodii* Δ*pyrE* or ∆*hydBA*, as described previously [35]. In both strains, ∆*hdcr* and *hydBA*/*hdcr*, the entire *hdcr* operon was deleted, leaving only the start codon of *fdhF1* and stop codon of *hydA2* in the chromosome. Plasmid pMTL_AW_KO_HDCR is based on pMTL84151 [36] from which the Gram-positive origin of replication was partially deleted by digestion of the vector with *Xmn*I and *Fsp*I following a blunt-end ligation. It further contains the *pyrE* cassette from *Eubacterium limosum* KIST612, consisting of the *pyrE* gene (ELI_0961) and 66 bp of its promoter region, as described before [28]. For targeting the *hdcr* gene cluster, a *hdcr* deletion cassette was cloned into the multiple cloning site, consisting of a 1256 bp upstream flanking region ending with the start codon of *fdhF1* and a 1085 bp downstream flanking region starting with the stop codon of *hydA2*. Both flanking regions were amplified via PCR, joined by splice-by-overlap-PCR (SOE-PCR) [37] and cloned into the plasmid using *Eco*RI and *Xba*I. A detailed description of transformation into *A. woodii* and integration of suicide plasmids for deletion of genes as well as recombination of the plasmid at its homologous regions towards the loss of genes has been published before [35]. After integration and exchange of the homologous regions of the *hdcr* operons in the *A. woodii* Δ*pyrE* strain and the Δ*hydBA* mutant, which lead to the loss of the 8567 bp of the *hdcr* operon, the deletion was verified by PCR and Sanger sequencing analysis [37].

### Complementation analyses

For the genetic complementation, plasmid pMTL84211_JM_Pptaack_hdcr was constructed. The plasmid is derived from pMTL84211 [36] and contains the seven *hdcr* genes from *A. woodii* (*fdhF1*, *hycB1*, *fdhF2*, *hycB2*, *fdhD*, *hycB3*, *hydA2*) downstream of a constitutive pta-ack promoter from *C. ljungdahlii*. Transformation of the plasmid into the Δ*hdcr* mutant was performed as described before [35] and the selection was carried out on agar plates with complex medium containing 20 mM fructose + 50 mM formate and 5 ng/µl clarithromycin. The presence of the plasmid in the complementation strain was verified by PCR and Sanger sequencing analysis [37]. Subsequently, the phenotypic complementation was analyzed by the growth experiments on fructose, H_2_ + CO_2_, glycine betaine and formate.

### Preparation of resting cells

Cells were cultivated on 50 mM glycine betaine in 0.5 to 2 l carbonate buffered complex medium to late exponential growth phase (OD_600_ of 0.3) and then harvested by centrifugation (Avanti J-25 and JA-10 Fixed-Angle Rotor; Beckman Coulter, Brea, CA, United States) at 8000 rpm and 4 °C for 10 min. The harvested cells were subsequently washed with 30 ml of buffer containing 50 mM imidazole (pH 7.0), 20 mM KCl, 20 mM MgSO_4_, 4 mM DTE and 4 µM resazurin by centrifugation at 8500 rpm and 4 °C for 10 min (Avanti J-25 and JA-25.50 Fixed-Angle Rotor; Beckman Coulter, Brea, CA, United States) and resuspended in 5 ml of imidazole buffer and kept in 16-ml Hungate tubes. All the steps were performed under strict anoxic conditions in an anoxic chamber (Coy Laboratory Products, Grass Lake, MI, United States) filled with N_2_/H_2_ (96–98%/2–4%; v/v). The total protein concentration in the resting cells was determined according to a previously described method [38].

### Cell suspension experiments

Cells were resuspended in 10 ml imidazole buffer (50 mM imidazole, 20 mM KCl, 20 mM NaCl, 20 mM MgSO_4_, 60 mM KHCO_3_, 4 mM DTE, 4 µM resazurin, pH 7.0) in 120-ml serum flasks to a total protein concentration of 1 mg/ml. As substrate, 50 mM glycine betaine, and if necessary, caffeate (4 mM) or H_2_ + CO_2_ was given. The suspension was incubated at 30 °C in water bath with shaking (150 rpm) and 0.5 ml samples were taken at each time point for determination of metabolites.

### Chemical analyses

The concentrations of H_2_ were determined by gas chromatography as described previously [39]. The samples for H_2_ determination were injected at 100 °C and separated on a ShinCarbon ST 80/100 column (2 m × 0.53 mm; Restek Corporation, Bellefonte, PA, USA). Nitrogen was used as the carrier gas with a head pressure of 400 kPa and a split flow of 30 ml/s. The samples were analyzed with a thermal conductivity detector at 100 °C and at an oven temperature of 40 °C. The concentrations of acetate and formate were determined by high-performance liquid chromatography [40]. For separation, a HyperREZ XP Carbohydrate H^+^ ion exchange column (Thermo Fisher Scientific, Waltham, MA, USA) was applied and degassed sulfuric acid (5 mM) was used as eluent at a flow rate of 0.6 ml/min. The oven was kept at 65 °C. 10 μl sample was injected by auto-sampler and analyzed with a refractive index detector (RefractoMax 520; Dionex, Sunnyvale, CA, USA) at 55 °C. Caffeate was determined photometrically at 312 nm using an extinction coefficient of 13.72 mM^−1^·cm^−1^ [26].

## Results

### Deletion of the *hdcr* operon

The *hdcr* operon in the thermophilic acetogen *T. kivui*, which does not grow on methyl groups, is essential for growth on H_2_ + CO_2_ but also for growth on glucose [41]; the latter was not expected since *T. kivui* has the genetic potential to grow by the conversion of glucose to 2 acetate, 2 CO_2_ and 4 H_2_, like *Thermotoga maritima* [42]. To analyse the role of the HDCR in *A. woodii*, we deleted the *hdcr* gene cluster through allelic exchange mutagenesis and verified the deletion by PCR and sequence analysis (Fig. S1).

### Methylotropic growth of the ∆*hdcr* mutant of *A. woodii*

**Fig. 2:**
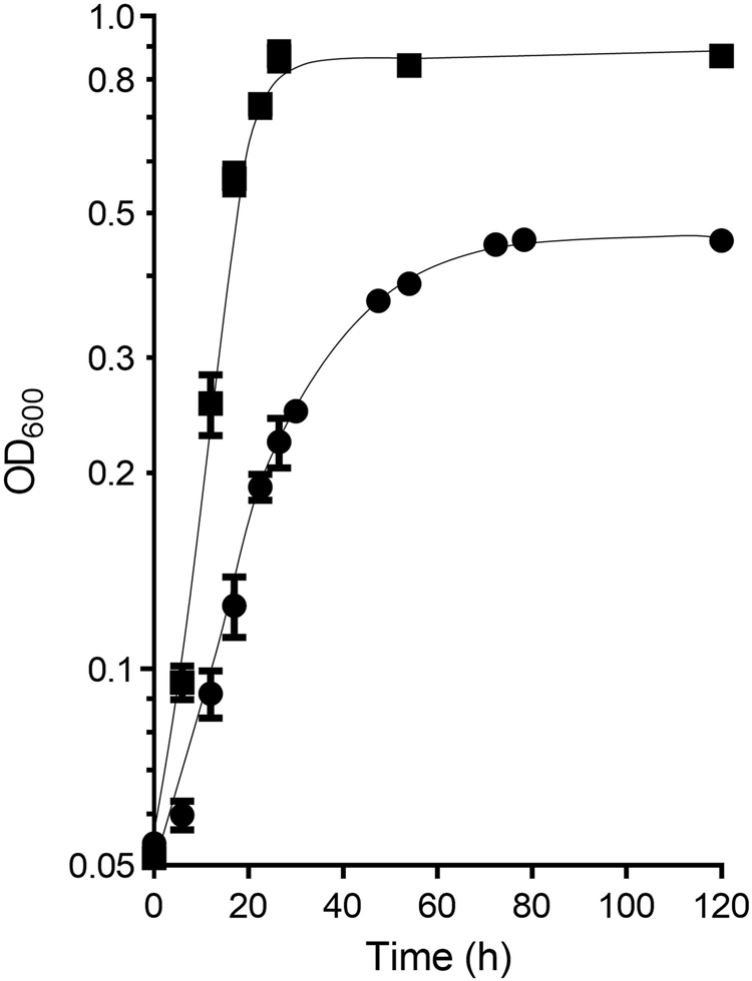
Growth of the Δ*hdcr* mutant on glycine betaine. The wild type (■) and the Δ*hdcr* mutant (●) were grown in 5 ml complex medium in 16-ml Hungate tubes at 30 °C with 50 mM glycine betaine as carbon and energy source. The growth experiments were performed in biological triplicates and each data point presents a mean with standard deviation (SD).

We tested growth of the ∆*hdcr* mutant on various compounds (Table S1). As seen with *T. kivui* [41], the mutant did not grow on H_2_ + CO_2_, but growth was restored by addition of formate, demonstrating that growth inhibition could be overcome by addition of the product of the HDCR reaction, formate. The same was observed with fructose. As in *T. kivui*, growth of the Δ*hdcr* mutant on fructose was not possible, demonstrating the essentiality of the WLP for redox disposal; however, growth was again restored by addition of formate as electron acceptor. We then concentrated on methyl group-containing substrates that are not utilized by *T. kivui*. *A. woodii* is well-known to utilize methyl group from methanol and other *O-*methyl group-containing substrates [6, 43]. While the wild type grew on methanol (60 mM), 3,4,5-trimethoxybenzoic acid (5 mM), 3,4-dimethoxybenzoic acid (5 mM), 3,4,5-trimethoxycinnamic acid (5 mM), 1,2-dimethoxybenzole (5 mM), vanillic acid (5 mM), isovanillic acid (5 mM), vanillin (5 mM) or ferulic acid (5 mM) as a sole substrate, the ∆*hdcr* mutant did not. However, the mutant doubled 1.2-times indicating little growth on components present in yeast extracts in low amounts. Previously, we identified the *N*-methyl group-containing substrate glycine betaine as a component of yeast extract that enables growth of *A. woodii* [7]. Indeed, growth of the mutant was possible on glycine betaine with a growth rate of 0.06 h^−1^ and to a final OD_600_ of 0.4, which is only 50% of the growth rate and final OD_600_ of the wild type strain (Fig. 2). We observed that the ∆*hdcr* mutant produced 10.4 ± 0.6 mM formate in addition to 21.2 ± 1.0 mM acetate, giving a molar ratio of formate to acetate of 1:2, in agreement with four electrons gained during oxidation of the methyl group to formate and two electrons required to reduce CO_2_ to CO. In contrast, the wild type strain performed homoacetogenesis, producing 35.5 ± 1.3 mM acetate, as was reported previously [7].

To verify that the phenotype observed was caused by deletion of the *hdcr* genes, the genes *fdhF1*, *hycB1*, *fdhF2*, *hycB2*, *fdhD*, *hycB3* and *hydA2* were cloned into the vector pMTL84211 under control of the constitutively expressed pta-ack promoter. After transformation into the ∆*hdcr* mutant, the transformant grew like the wild type on fructose, H_2_ + CO_2_, glycine betaine, and formate (Fig. S2), excluding polar effects of the deletion on downstream regions.

### Formatogenesis in resting cells of the ∆*hdcr* mutant

**Fig. 3:**
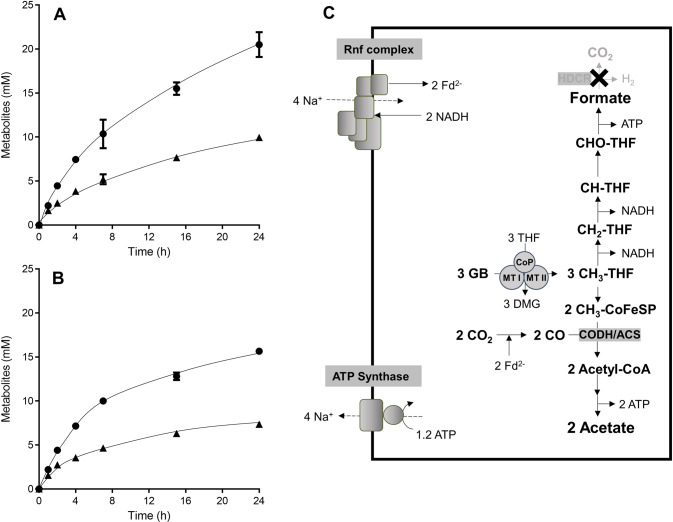
Formatogenesis from glycine betaine in the Δ*hdcr* mutant of *A. woodii*. Cells of the (**A**) Δ*hdcr* and (**B**) Δ*hydBA/hdcr* mutants were grown in complex media with 50 mM glycine betaine and harvested in the early stationary growth phase. After washing, the cells were resuspended in 10 ml of cell suspension buffer in 120-ml serum flasks under a N_2_/CO_2_ atmosphere at a total protein concentration of 1 mg/ml. 50 mM glycine betaine was given to the cell suspensions as carbon and energy source. Acetate (●) and formate (▲) were determined at each time point. Each data point presents a mean ± SD; *n* =  2 independent biological replicates. **C** Biochemistry and bioenergetics of formatogenesis. GB glycine betaine, DMG dimethylglycine, Fd ferredoxin, THF tetrahydrofolate, CODH/ACS CO dehydrogenase/acetyl coenzyme A synthase, CoFeSP corrinoid iron-sulfur protein, MTI methyltransferase I, MTII methyltransferase II, CoP corrinoid protein. The stoichiometry of the ATP synthase is 3.3 Na^+^/ATP [67] and for the Rnf complex a stoichiometry of 2 Na^+^/2 e^−^ is assumed.

We further analyzed carbon flow and products produced from methyl groups in resting cells of the ∆*hdcr* mutant. For these experiments, cells were grown on 50 mM glycine betaine until stationary growth phase and after harvesting, cell suspensions were prepared. When 50 mM glycine betaine was given as a substrate, resting cells of the ∆*hdcr* mutant produced 10.0 ± 0.4 mM formate and 20.4 ± 1.4 mM acetate with a formate/acetate ratio of 1:2, which corresponds to the fermentation balance in growing cells. The production rate of formate and acetate showed a ratio of 1:2 as well (7.6 ± 0.7 nmol min^−1^ mg^−1^ and 16.1 ± 1.0 nmol min^−1^ mg^−1^, respectively) (Fig. 3A). In contrast, wild type cells performed homoacetogenesis, producing 33.0 ± 1.5 mM acetate (Fig. S3). We then determined the fermentation balance of the ∆*hdcr* mutant in the absence of NaCl to reveal whether the Rnf complex is required for formatogenesis during methylotrophic fermentation in the ∆*hdcr* mutant. In the absence of NaCl, glycine betaine was not converted to formate and acetate, indicating that the Rnf complex is essential for formatogenesis, most likely to reoxidize NADH, with concomitant reduction of ferredoxin (cf. Fig. 1).

Oxidation of methyl groups to CO_2_ produces molecular hydrogen that is then oxidized by the electron-bifurcating hydrogenase with concomitant reduction of NAD^+^ and ferredoxin. Since only reduced ferredoxin but not NADH is required as reductant in this metabolic scenario, NADH is oxidized with reduction of ferredoxin by the Rnf complex (cf. Fig. 1). To determine whether the electron bifurcating hydrogenase is required for formatogenesis from glycine betaine, we repeated the cell suspension experiments with the ∆*hydBA/hdcr* mutant which lacks genes encoding the HDCR and the electron bifurcating hydrogenase (Fig. 3B). Resting cells of the ∆*hydBA/hdcr* mutant produced 7.4 ± 0.2 mM formate and 15.7 ± 0.2 mM acetate, showing that the bifurcating hydrogenase is not involved in formatogenesis from glycine betaine in the ∆*hdcr* mutant. In agreement with this notion is the observed growth of the ∆*hydBA/hdcr* double mutant on glycine betaine (Fig. S4). The ∆*hydBA* mutant [28] also grew on glycine betaine and also produced formate, but less than the ∆*hydBA/hdcr* or ∆*hdcr* mutant. We observed that the ∆*hydBA* mutant produced molecular hydrogen (produced by the HDCR enyzme) and growth of the mutant was inhibited by addition of molecular hydrogen.

The experiments described above are in agreement with the following hypothesis (Fig. 3C): the methyl group from glycine betaine is oxidized to formate, generating 2 NADH and 1 ATP, and the 2 NADH are oxidized by the Rnf complex coupled to the reduction of 2 ferredoxins; the Rnf complex operates in reverse, driven by the electrochemical Na^+^ potential across the membrane, established by the hydrolysis of 1.2 ATP [23]. Then, 2 external CO_2_ are reduced to 2 CO and converted to 2 acetyl-CoA with 2 methyl groups from glycine betaine. In sum, one formate and 2 acetate are produced from 3 glycine betaine in the ∆*hdcr* mutant (Eq. 2):

2 $$	3\;\left( {{{{{{{{\mathrm{CH}}}}}}}}_{{{{{{{\mathrm{3}}}}}}}}} \right)_3{{{{{{{\mathrm{ - N}}}}}}}}^ + {{{{{{{\mathrm{ - CH}}}}}}}}_{{{{{{{\mathrm{2}}}}}}}}{{{{{{{\mathrm{COOH}}}}}}}}+ 2\;{{{{{{{\mathrm{CO}}}}}}}}_{{{{{{{\mathrm{2}}}}}}}} \to {{{{{{{\mathrm{HCOOH}}}}}}}} + 2\;{{{{{{{\mathrm{CH}}}}}}}}_{{{{{{{\mathrm{3}}}}}}}}{{{{{{{\mathrm{COOH}}}}}}}} \\ 	 + 3\;\left( {{{{{{{{\mathrm{CH}}}}}}}}_{{{{{{{\mathrm{3}}}}}}}}} \right)_2{{{{{{{\mathrm{ - N}}}}}}}} {{{{{{{\mathrm{ - CH}}}}}}}}_{{{{{{{\mathrm{2}}}}}}}}{{{{{{{\mathrm{COOH}}}}}}}} + 1.8\;{{{{{{{\mathrm{ATP}}}}}}}}$$

This metabolism yields 0.6 mol of ATP per mol of methyl group.

### Addition of H_2_ abolishes formatogenesis from glycine betaine

**Fig. 4:**
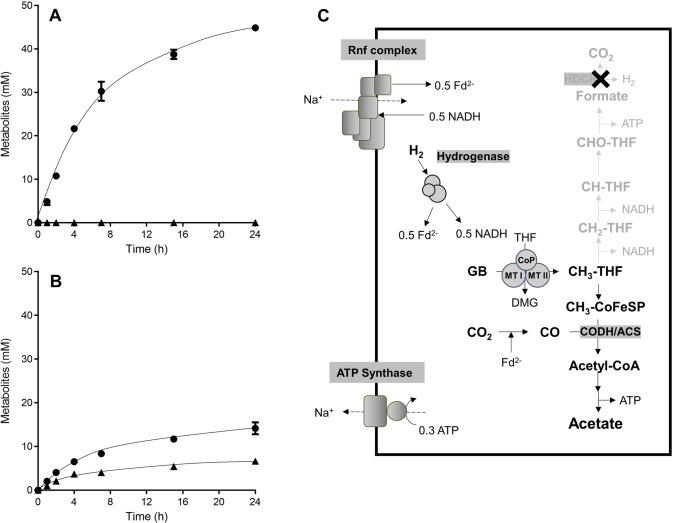
Formatogenesis from glycine betaine is abolished in the Δ*hdcr* mutant by H_2_. Cells of the (**A**) Δ*hdcr* and (**B**) Δ*hydBA/hdcr* mutants were grown in complex media with 50 mM glycine betaine and harvested in the early stationary growth phase. After washing, the cells were resuspended in 10 ml of cell suspension buffer in 120-ml serum flasks under a N_2_/CO_2_ atmosphere at a total protein concentration of 1 mg/ml. 50 mM glycine betaine and 1 bar of H_2_ + CO_2_ (80:20, v/v) was given to the cell suspensions as carbon and energy source. Acetate (●) and formate (▲) were determined at each time point. Each data point presents a mean ± SD; *n* =  2 independent biological replicates. **C** Biochemistry and bioenergetics of acetogenesis from glycine betaine + H_2_ + CO_2_. GB glycine betaine, DMG dimethylglycine, Fd ferredoxin, THF tetrahydrofolate, CODH/ACS CO dehydrogenase/acetyl coenzyme A synthase, CoFeSP corrinoid iron-sulfur protein. MTI methyltransferase I, MTII methyltransferase II, CoP corrinoid protein. The stoichiometry of the ATP synthase is 3.3 Na^+^/ATP [67] and for the Rnf complex a stoichiometry of 2 Na^+^/2 e^−^ is assumed.

The presence of molecular hydrogen abolishes methyl group oxidation in methanogens [44] as well as acetogens [25] and redirects carbon exclusively to methane or acetate, respectively. In the presence of H_2_, resting cells of the ∆*hdcr* mutant did not produce formate anymore (Fig. 4A). Instead, 44.9 ± 0.6 mM acetate was produced from 50 mM glycine betaine with an acetate/glycine betaine ratio of 0.9. The acetate formation rate was 40.6 ± 2.4 nmol min^−1^ mg^−1^ which was 2.5 times faster than that in resting cells in the absence of H_2_. In the absence of NaCl, acetate was not produced, implying that the Rnf complex is required for this metabolism. We repeated these experiments with resting cells of the ∆*hydBA/hdcr* mutant to experimentally verify the assumption that the bifurcating hydrogenase is essential to revert the metabolism of the *hdcr* mutant to homoacetogenesis in the presence of H_2_. Indeed, resting cells of the ∆*hydBA/hdcr* mutant still produced 6.7 ± 0.4 mM formate and 14.2 ± 1.4 mM acetate even in the presence of H_2_ (Fig. 4B), indicating that H_2_ needs to be converted to reduced ferredoxin and NADH to abolish formatogenesis.

In sum, in the presence of H_2_, one H_2_ is converted to 0.5 NADH and 0.5 reduced ferredoxin by the electron bifurcating hydrogenase and 0.5 NADH is further oxidized via the Rnf complex to give 0.5 reduced ferredoxin, coupled with the hydrolysis of 0.3 ATP (Fig. 4C). Then, one reduced ferredoxin is utilized to reduce CO_2_ to CO that is condensed to acetyl-CoA with a methyl-THF derived from glycine betaine and CoA. Overall, one acetate is produced from one glycine betaine in the presence of H_2_ in the ∆*hdcr* mutant and formatogenesis is completely abolished (Eq. 3):

3 $$	{{{{{{{\mathrm{H}}}}}}}}_{{{{{{{\mathrm{2}}}}}}}} + {{{{{{{\mathrm{CO}}}}}}}}_{{{{{{{\mathrm{2}}}}}}}} + \left( {{{{{{{{\mathrm{CH}}}}}}}}_{{{{{{{\mathrm{3}}}}}}}}} \right)_3{{{{{{{\mathrm{ - N}}}}}}}}^ + {{{{{{{\mathrm{ - CH}}}}}}}}_{{{{{{{\mathrm{2}}}}}}}}{{{{{{{\mathrm{COOH}}}}}}}} \to {{{{{{{\mathrm{CH}}}}}}}}_{{{{{{{\mathrm{3}}}}}}}}{{{{{{{\mathrm{COOH}}}}}}}} \\ 	 + \left( {{{{{{{{\mathrm{CH}}}}}}}}_{{{{{{{\mathrm{3}}}}}}}}} \right)_2{{{{{{{\mathrm{ - N}}}}}}}} {{{{{{{\mathrm{ - CH}}}}}}}}_{{{{{{{\mathrm{2}}}}}}}}{{{{{{{\mathrm{COOH}}}}}}}} + 0.7\;{{{{{{{\mathrm{ATP}}}}}}}}$$

This metabolism yields 0.7 mol of ATP per mol of methyl group.

### The ∆*hdcr* mutant performs exclusively formatogenesis from glycine betaine in the presence of caffeate

**Fig. 5:**
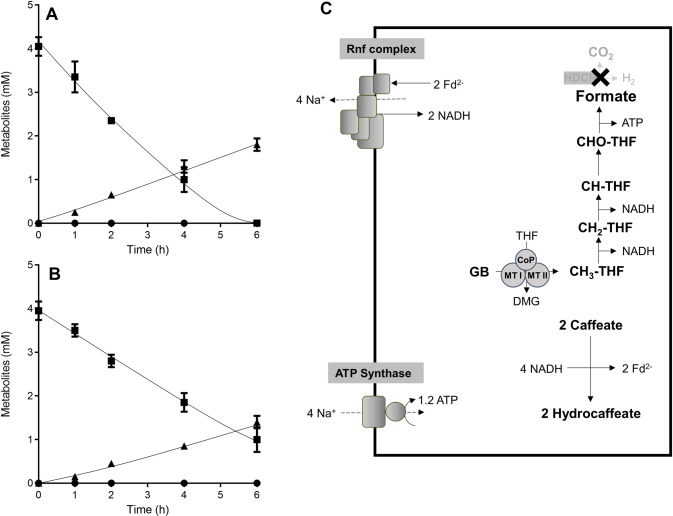
Homoformatogenesis from glycine betaine in the Δ*hdcr* mutant of *A. woodii* in the presence of caffeate. Cells of the (**A**) Δ*hdcr* and (**B**) Δ*hydBA/hdcr* mutants were grown in complex media with 50 mM glycine betaine + 2 mM caffeate and harvested in the early stationary growth phase. After washing, the cells were resuspended in 10 ml of cell suspension buffer in 120-ml serum flasks under a N_2_ atmosphere at a total protein concentration of 1 mg/ml. 2 mM glycine betaine + 4 mM caffeate was given to the cell suspensions as carbon and energy source. Acetate (●), formate (▲) and caffeate (■) were determined at each time point. Each data point presents a mean ± SD; *n* =  2 independent biological replicates. **C** Biochemistry and bioenergetics of homoformatogenesis from glycine betaine and caffeate. GB glycine betaine, DMG dimethylglycine, Fd ferredoxin, THF tetrahydrofolate, CODH/ACS CO dehydrogenase/acetyl coenzyme A synthase, CoFeSP corrinoid iron-sulfur protein, MTI methyltransferase I, MTII methyltransferase II, CoP corrinoid protein. The stoichiometry of the ATP synthase is 3.3 Na^+^/ATP [67] and for the Rnf complex a stoichiometry of 2 Na^+^/2 e^−^ is assumed. Caffeate is first activated to caffeyl-CoA [68] and then reduced to hydrocaffeyl-CoA by the electron bifurcating caffeyl-CoA reductase, also yielding 2 reduced ferredoxin.

The experiments described above showed that it is possible to reprogram carbon flow from methyl groups to formate, but CO_2_ reduction to CO followed by acetate formation is still required for redox balancing. In previous studies, *A. woodii* performed exclusively oxidative conversion of methanol to CO_2_ in the presence the alternative electron acceptor caffeate [25, 26, 45]. In the wild type, caffeate was reduced to hydrocaffeate but acetate was no longer produced. In the same manner, we expected that the ∆*hdcr* mutant should perform homoformatogenesis from glycine betaine in the presence of caffeate. Indeed, resting cells of the ∆*hdcr* mutant oxidized the methyl group of glycine betaine stoichiometrically to formate and reduced caffeate to hydrocaffeate but did not produce acetate (Fig. 5A). 1.8 ± 0.1 mM formate was produced from 2 mM glycine betaine in the presence of 4 mM caffeate with a glycine betaine:formate ratio of 1:0.9. The same was true in resting cells of the ∆*hydBA/hdcr* mutant indicating that hydrogenase is not involved in formatogenesis (Fig. 5B). Consistently, the ∆*hdcr* and the ∆*hydBA/hdcr* mutant grew on glycine betaine and caffeate (Fig. S5) and performed homoformatogenesis as well, generating 1.6 ± 0.1 mM formate from 2 mM glycine betaine + 4 mM caffeate with a glycine betaine:formate ratio of 1:0.8.

In sum, one glycine betaine is oxidized to formate generating 2 NADH and one ATP (Fig. 5C). To reduce 2 caffeate by the electron bifurcating caffeyl-CoA reductase, 4 NADH are oxidized, and two ferredoxins are reduced as well. Then, the 2 reduced ferredoxins are oxidized to generate 2 NADH by the Rnf complex, followed by the synthesis of 1.2 ATP. In sum, the ∆*hdcr* mutant produces one formate from one glycine betaine in the presence of 2 caffeate (Eq. 4):

4 $$	\left( {{{{{{{{\mathrm{CH}}}}}}}}_{{{{{{{\mathrm{3}}}}}}}}} \right)_3{{{{{{{\mathrm{ - N}}}}}}}}^ + {{{{{{{\mathrm{ - CH}}}}}}}}_{{{{{{{\mathrm{2}}}}}}}}{{{{{{{\mathrm{COOH}}}}}}}} + 2\;{{{{{{{\mathrm{caffeate}}}}}}}} \to {{{{{{{\mathrm{HCOOH}}}}}}}} + 2\;{{{{{{{\mathrm{hydrocaffeate}}}}}}}}\\ 	 + \left( {{{{{{{{\mathrm{CH}}}}}}}}_{{{{{{{\mathrm{3}}}}}}}}} \right)_2{{{{{{{\mathrm{ - N}}}}}}}} {{{{{{{\mathrm{ - CH}}}}}}}}_{{{{{{{\mathrm{2}}}}}}}}{{{{{{{\mathrm{COOH}}}}}}}} + 2.2\;{{{{{{{\mathrm{ATP}}}}}}}}$$

This metabolism yields 2.2 mol of ATP per mol of methyl group.

## Discussion

The hallmark of acetogenic bacteria is the production of acetate from two molecules of CO_2_ via the WLP. However, it has been noted that some species naturally produce ethanol, butyrate, succinate or even higher carbon chain compounds from the central intermediate of the WLP, acetyl-CoA [46–50]. The intermediates of the WLP have not yet been considered as catabolic end products. The formation of methylated compounds such as, for example, methyl sulfide from H_2_ + CO_2_ is thermodynamically unfeasible, because it results in a net ATP loss. However, it could be produced as a side product, but this has not been reported. This is contrast to formate, a known intermediate of the WLP. Transient production of formate has been observed in different acetogenic species [31–33], it is excreted in small amounts into the medium and later taken up again. *A. woodii* has two genes encoding a formyl-THF synthetase, *fhs1* (Awo_c09260) and *fhs2* (Awo_c08040) [15]; *fhs2* forms an operon with a gene encoding a formate transporter (*fdhC*, Awo_c08050) [33]. Deletion of the *fhs2/fdhC* genes led to a complete loss of transient formate production, indicating their involvement in formate production. However, formate is produced as a side product, most likely because the formyl-THF synthetase reaction is the kinetic bottleneck upon entry of formate into the THF pathway; formate production is not energy conserving.

### Energetics of formate production

The WLP is reversible and works in the oxidative direction during syntrophic acetate oxidation [51] as well as methyl group oxidation. Formation of CO_2_ and H_2_ from a methyl group (for example, methanol) is endergonic (Eq. 5):

5 $${{{{{{{\mathrm{CH}}}}}}}}_{{{{{{{\mathrm{3}}}}}}}}{{{{{{{\mathrm{OH}}}}}}}} + 2\;{{{{{{{\mathrm{H}}}}}}}}_{{{{{{{\mathrm{2}}}}}}}}{{{{{{{\mathrm{O}}}}}}}} \to {{{{{{{\mathrm{HCO}}}}}}}}_{{{{{{{\mathrm{3}}}}}}}}^ - + 3\;{{{{{{{\mathrm{H}}}}}}}}_{{{{{{{\mathrm{2}}}}}}}} + {{{{{{{\mathrm{H}}}}}}}}^{{{{{{{\mathrm{ + }}}}}}}}\;\;\;\;\Delta {{{{{{{{\mathrm{G}}}}}}}}_{0}}^\prime = + 23.5\;{{{{{{{\mathrm{kJ/mol}}}}}}}}$$

The same is true for formate formation from methanol, according to Eq. 6.

6 $${{{{{{{\mathrm{CH}}}}}}}}_{{{{{{{\mathrm{3}}}}}}}}{{{{{{{\mathrm{OH}}}}}}}} + {{{{{{{\mathrm{H}}}}}}}}_{{{{{{{\mathrm{2}}}}}}}}{{{{{{{\mathrm{O}}}}}}}} \to {{{{{{{\mathrm{HCOO}}}}}}}}^{{{{{{{\mathrm{ - }}}}}}}} + 2\;{{{{{{{\mathrm{H}}}}}}}}_{{{{{{{\mathrm{2}}}}}}}} + {{{{{{{\mathrm{H}}}}}}}}^{{{{{{{\mathrm{ + }}}}}}}}\;\;\;\;\Delta {{{{{{{{\mathrm{G}}}}}}}}_{0}}^\prime = + 21.8\;{{{{{{{\mathrm{kJ/mol}}}}}}}}$$

At a H_2_ pressure of 1 atm, reactions according to Eqs. 5 and 6 are endergonic. However, if the ∆G’ values are calculated for H_2_ concentrations of 10^−6^ to 10^−3 ^atm [27, 52, 53], the oxidation reaction becomes energetically possible (∆G’ = −77.2 to −27.8 kJ/mol). Low hydrogen concentrations are maintained in the natural habitat of acetogens by hydrogen-consuming partners such as sulfate reducers or methanogens [53, 54]. Indeed, when *A. woodii* was grown in co-culture with a methanogen, fructose was oxidized to 2 acetate, 2 CO_2_ and 4 H_2_, the latter was used by the methanogen to produce methane [29]. When methanol was used as a substrate, it was mainly oxidized to CO_2_ + H_2_ that were removed by the methanogen [30]. At a hydrogen pressure of 0.04 atm, the reaction is at equilibrium.

Methanol oxidation in *A. woodii* involves the methylene-THF reductase and the methylene-THF dehydrogenase reaction, both of which are coupled to the reduction of NAD^+^. The greatest energetic barrier is the production of hydrogen. Electron transfer from NADH (E_0_’ = −320 mV) to H^+^ (E_0_’ = −414 mV) is endergonic and driven by simultaneous electron transfer from reduced ferredoxin (E_0_’ ≈ −450 mV) to H^+^. Whereas the latter reaction is exergonic with −6.9 kJ/mol, the former is endergonic with 18.5 kJ/mol, making the overall reaction endergonic with 11.6 kJ/mol. The reaction would be at equilibrium at a hydrogen partial pressure of 0.1 atm, lower concentrations making the reaction thermodynamically possible. A maximum of 0.4 ATP/mol of methyl group can be obtained mechanistically, one in the formyl-THF synthetase reaction and 0.6 ATP has to be invested in the ATPase/Rnf reaction to transfer 2 electrons from NADH to ferredoxin (Fig. S6). The same calculations can be done for formate production: at a formate concentration of 150 µM, formate production is in equilibrium. Any lower value would make the reaction feasible, allowing also for a maximum of 0.4 ATP/mol of methyl group.

### Ecological implications

Energetically, the oxidation of a methyl group to H_2_ + CO_2_ or formate does not make a big difference and the demonstrated capability of *A. woodii* to grow on methyl groups by formatogenesis does not necessarily reflect that this will occur in nature. Indeed, this seems rather unlikely since *A. woodii* has the HDCR enzyme that oxidizes formate to H_2_ + CO_2_ with unprecedented rates [16, 17]. However, certain environmental conditions such as high hydrogen and/or CO_2_/bicarbonate concentrations, or a lack of molybdenum or iron may disfavor formate oxidation but favor formate production. Reversal of the WLP also occurs in syntrophic oxidation of acetate [55–57]. Some bacteria such as *Thermoacetogenium phaeum* [58, 59] and *Tepidanaerobacter acetatoxydans* [60, 61] can switch from acetogenic to acetoclastic lifestyle when growing with a hydrogen-oxidizing partner, i. e. the methyl group of acetate is oxidized as the methyl group of glycine betaine or methanol. However, *T. acetatoxydans* does not have a formate dehydrogenase arguing for formate transfer between this acetogen and methanogens during syntrophic acetate oxidation [61]. Moreover, most acetogenic species of the genus *Blautia* lack genes encoding a HDCR or formate dehydrogenase [62]. Since *Blautia* species are active members of the gut microbiome [63], formate may be an end product of acetogenic metabolism and transferred to methanogens as carbon and energy source. Thus, our data do not only provide a genetic proof for formate as end product of methyl group oxidation via the WLP but opens the way to study the differences and advantages of formate and hydrogen as electron carrier in syntrophic associations.

Formate and H_2_ are energetically equivalent and can be transformed into one another by soluble or membrane-bound enzymes. Since the equilibrium constant is almost one, the direction is controlled by the concentration of the reductants. Formate production may be favored in H_2_-rich ecosystems and electrons shuttled to the electron consuming partners via formate, not hydrogen. Such ecosystems are, for example, deep sea hydrothermal vents in which serpentinization leads to hydrogen production [64, 65]. *Thermococcus paralvinellae* isolated from a deep sea vent is a chemosyntroph that produces H_2_ from H^+^ in the absence of sulfur [66]; H_2_ is then used by the *Methanococci*. At high hydrogen pressures, H_2_ formation is inhibited, and formate is produced from H_2_ + CO_2_ by the formate hydrogen lyase in *T. paralvinellae* [64]. Formate is then used by the *Methanococci* as carbon and electron source.

Formate production gives cells a growth advantage when H_2_ is inhibiting [65]. This is observed in hydrothermal vents as well as in “modern day” anoxic environments indicating that it is an old mechanism to ameliorate H_2_ inhibition. Whether or not natural formatogenic lifestyle exists in acetogens, remains to be elucidated.

## Supplementary information

Supplementary Information

## Data Availability

The data generated in this study are available from the corresponding author on reasonable request.

## References

[1] Hippe H, Caspari D, Fiebig K, Gottschalk G (1979). Utilization of trimethylamine and other N-methyl compounds for growth and methane formation by *Methanosarcina barkeri*. Proc Natl Acad Sci USA.

[2] Ranalli G, Whitmore TN, Lloyd MD (1986). Methanogenesis from methanol in *Methanosarcina barkeri* studied using membrane inlet mass spectrometry. FEMS Microbiol Lett.

[3] Braun M, Stolp H (1985). Degradation of methanol by a sulfate reducing bacterium. Arch Microbiol.

[4] Nanninga HJ, Gottschal JC (1986). Microbial problems with waste from potato-starch processing. Microbiol Sci.

[5] Sousa DZ, Visser M, van Gelder AH, Boeren S, Pieterse MM, Pinkse MWH (2018). The deep-subsurface sulfate reducer *Desulfotomaculum kuznetsovii* employs two methanol-degrading pathways. Nat Commun.

[6] Kremp F, Poehlein A, Daniel R, Müller V (2018). Methanol metabolism in the acetogenic bacterium *Acetobacterium woodii*. Environ Microbiol.

[7] Lechtenfeld M, Heine J, Sameith J, Kremp F, Müller V (2018). Glycine betaine metabolism in the acetogenic bacterium *Acetobacterium woodii*. Environ Microbiol.

[8] Kremp F, Müller V (2021). Methanol and methyl group conversion in acetogenic bacteria: biochemistry, physiology and application. FEMS Microbiol Rev.

[9] Stupperich E, Konle R (1993). Corrinoid-dependent methyl transfer reactions are involved in methanol and 3,4-dimethoxybenzoate metabolism by *Sporomusa ovata*. Appl Environ Microbiol.

[10] Kreft JU, Schink B (1994). *O*-demethylation by the homoacetogenic anaerobe *Holophaga foetida* studied by a new photometric methylation assay using electrochemically produced cobalamin. Eur J Biochem.

[11] Kaufmann F, Wohlfarth G, Diekert G (1998). *O*-demethylase from *Acetobacterium dehalogenans*-substrate specificity and function of the participating proteins. Eur J Biochem.

[12] Engelmann T, Kaufmann F, Diekert G (2001). Isolation and characterization of a veratrol:corrinoid protein methyl transferase from *Acetobacterium dehalogenans*. Arch Microbiol.

[13] Siebert A, Schubert T, Engelmann T, Studenik S, Diekert G (2005). Veratrol-O-demethylase of *Acetobacterium dehalogenans*: ATP-dependent reduction of the corrinoid protein. Arch Microbiol.

[14] Naidu D, Ragsdale SW (2001). Characterization of a three-component vanillate *O*-demethylase from *Moorella thermoacetica*. J Bacteriol.

[15] Poehlein A, Schmidt S, Kaster A-K, Goenrich M, Vollmers J, Thürmer A (2012). An ancient pathway combining carbon dioxide fixation with the generation and utilization of a sodium ion gradient for ATP synthesis. PLoS One.

[16] Schuchmann K, Müller V (2013). Direct and reversible hydrogenation of CO_2_ to formate by a bacterial carbon dioxide reductase. Science.

[17] Kottenhahn P, Schuchmann K, Müller V (2018). Efficient whole cell biocatalyst for formate-based hydrogen production. Biotechnol Biofuels.

[18] Dietrich HM, Righetto RD, Kumar A, Wietrzynski W, Trischler R, Schuller SK (2022). Membrane-anchored HDCR nanowires drive hydrogen-powered CO_2_ fixation. Nature.

[19] Ragsdale SW, Ljungdahl LG (1984). Purification and properties of NAD-dependent 5,10-methylenetetrahydrofolate dehydrogenase from *Acetobacterium woodii*. J Biol Chem.

[20] Bertsch J, Öppinger C, Hess V, Langer JD, Müller V (2015). Heterotrimeric NADH-oxidizing methylenetetrahydrofolate reductase from the acetogenic bacterium *Acetobacterium woodii*. J Bacteriol.

[21] Ragsdale SW, Ljungdahl LG, Dervartanian DV (1983). Isolation of carbon monoxide dehydrogenase from *Acetobacterium woodii* and comparison of its properties with those of the *Clostridium thermoaceticum* enzyme. J Bacteriol.

[22] Shanmugasundaram T, Ragsdale SW, Wood HG (1988). Role of carbon monoxide dehydrogenase in acetate synthesis by the acetogenic bacterium, *Acetobacterium woodii*. BioFactors.

[23] Hess V, Schuchmann K, Müller V (2013). The ferredoxin:NAD^+^ oxidoreductase (Rnf) from the acetogen *Acetobacterium woodii* requires Na^+^ and is reversibly coupled to the membrane potential. J Biol Chem.

[24] Schuchmann K, Müller V (2012). A bacterial electron bifurcating hydrogenase. J Biol Chem.

[25] Litty D, Kremp F, Muller V (2022). One substrate, many fates: different ways of methanol utilization in the acetogen *Acetobacterium woodii*. Environ Microbiol.

[26] Dilling S, Imkamp F, Schmidt S, Müller V (2007). Regulation of caffeate respiration in the acetogenic bacterium *Acetobacterium woodii*. Appl Environ Microbiol.

[27] Schink B, Montag D, Keller A, Müller N (2017). Hydrogen or formate: alternative key players in methanogenic degradation. Environ Microbiol Rep.

[28] Wiechmann A, Ciurus S, Oswald F, Seiler VN, Müller V (2020). It does not always take two to tango: “Syntrophy” via hydrogen cycling in one bacterial cell. ISME J.

[29] Winter JU, Wolfe RS (1980). Methane formation from fructose by syntrophic associations of *Acetobacterium woodii* and different strains of methanogens. Arch Microbiol.

[30] Heijthuijsen JHFG, Hansen TA (1986). Interspecies hydrogen transfer in co-cultures of methanol-utilizing acidogens and sulfate-reducing or methanogenic bacteria. FEMS Microbiol Ecol.

[31] Peters V, Janssen PH, Conrad R (1999). Transient production of formate during chemolithotrophic growth of anaerobic microorganisms on hydrogen. Curr Microbiol.

[32] Oswald F, Stoll IK, Zwick M, Herbig S, Sauer J, Boukis N (2018). Formic acid formation by *Clostridium ljungdahlii* at elevated pressures of carbon dioxide and hydrogen. Front Bioeng Biotechnol.

[33] Moon J, Dönig J, Kramer S, Poehlein A, Daniel R, Müller V (2021). Formate metabolism in the acetogenic bacterium *Acetobacterium woodii*. Environ Microbiol.

[34] Heise R, Müller V, Gottschalk G (1989). Sodium dependence of acetate formation by the acetogenic bacterium *Acetobacterium woodii*. J Bacteriol.

[35] Westphal L, Wiechmann A, Baker J, Minton NP, Müller V (2018). The Rnf complex is an energy coupled transhydrogenase essential to reversibly link cellular NADH and ferredoxin pools in the acetogen *Acetobacterium woodii*. J Bacteriol.

[36] Heap JT, Pennington OJ, Cartman ST, Minton NP (2009). A modular system for *Clostridium* shuttle plasmids. J Microbiol Methods.

[37] Sanger FS, Nickelen F, Coulson AR (1977). DNA-sequencing with chain-terminating inhibitors. Proc Natl Acad Sci USA.

[38] Schmidt K, Liaaen-Jensen S, Schlegel HG (1963). Die Carotinoide der *Thiorhodaceae*. Arch Mikrobiol.

[39] Weghoff MC, Müller V (2016). CO metabolism in the thermophilic acetogen *Thermoanaerobacter kivui*. Appl Environ Microbiol.

[40] Moon J, Henke L, Merz N, Basen M (2019). A thermostable mannitol-1-phosphate dehydrogenase is required in mannitol metabolism of the thermophilic acetogenic bacterium *Thermoanaerobacter kivui*. Environ Microbiol.

[41] Jain S, Dietrich HM, Müller V, Basen M (2020). Formate is required for growth of the thermophilic acetogenic bacterium *Thermoanaerobacter kivui* lacking hydrogen-dependent carbon dioxide reductase (HDCR). Front Microbiol.

[42] Schröder C, Selig M, Schönheit P (1994). Glucose fermentation to acetate, CO_2_ and H_2_ in the anaerobic hyperthermophilic eubacterium *Thermotoga maritima* - involvement of the Embden-Meyerhof pathway. Arch Microbiol.

[43] Bache R, Pfennig N (1981). Selective isolation of *Acetobacterium woodii* on methoxylated aromatic acids and determination of growth yields. Arch Microbiol.

[44] Müller V, Blaut M, Gottschalk G (1986). Utilization of methanol plus hydrogen by *Methanosarcina barkeri* for methanogenesis and growth. Appl Environ Microbiol.

[45] Tschech A, Pfennig N (1984). Growth yield increase linked to caffeate reduction in *Acetobacterium woodii*. Arch Microbiol.

[46] Matthies C, Freiberger A, Drake HL (1993). Fumarate dissimilation and differential reductant flow by *Clostridium formicoaceticum* and *Clostridium aceticum*. Arch Microbiol.

[47] Misoph M, Drake HL (1996). Effect of CO_2_ on the fermentation capacities of the acetogen *Peptostreptococcus productus* U-1. J Bacteriol.

[48] Köpke M, Held C, Hujer S, Liesegang H, Wiezer A, Wollherr A (2010). *Clostridium ljungdahlii* represents a microbial production platform based on syngas. Proc Natl Acad Sci USA.

[49] Litty D, Müller V (2021). Butyrate production in the acetogen *Eubacterium limosum* is dependent on the carbon and energy source. Micro Biotechnol.

[50] Doyle DA, Smith PR, Lawson PA, Tanner RS. *Clostridium muellerianum* sp. nov., a carbon monoxide-oxidizing acetogen isolated from old hay. Int J Syst Evol Microbiol. 2022;72. 10.1099/ijsem.0.005297 35353674

[51] Keller A, Schink B, Muller N (2019). Energy-conserving enzyme systems active during syntrophic acetate oxidation in the thermophilic bacterium *Thermacetogenium phaeum*. Front Microbiol.

[52] Hungate RE (1967). Hydrogen as an intermediate in the rumen fermentation. Arch Mikrobiol.

[53] Thauer RK, Jungermann K, Decker K (1977). Energy conservation in chemotrophic anaerobic bacteria. Bacteriol Rev.

[54] Bryant MP, Campbell LL, Reddy CA, Crabill MR (1977). Growth of *Desulfovibrio* in lactate or ethanol media low in sulfate in association with H_2_-utilizing methanogenic bacteria. Appl Environ Microbiol.

[55] Schink B, Stams AJM. Syntrophism among prokaryotes. In: Rosenberg E, DeLong EF, Lory S, Stackebrandt E, Thompson F, editors. The prokaryotes. Berlin, Heidelberg: Springer; 2013. p. 471–93.

[56] McInerney MJ, Struchtemeyer CG, Sieber J, Mouttaki H, Stams AJ, Schink B (2008). Physiology, ecology, phylogeny, and genomics of microorganisms capable of syntrophic metabolism. Ann N Y Acad Sci.

[57] McInerney MJ, Sieber JR, Gunsalus RP (2009). Syntrophy in anaerobic global carbon cycles. Curr Opin Biotechnol.

[58] Hattori S, Galushko AS, Kamagata Y, Schink B (2005). Operation of the CO dehydrogenase/acetyl coenzyme A pathway in both acetate oxidation and acetate formation by the syntrophically acetate-oxidizing bacterium *Thermacetogenium phaeum*. J Bacteriol.

[59] Oehler D, Poehlein A, Leimbach A, Müller N, Daniel R, Gottschalk G (2012). Genome-guided analysis of physiological and morphological traits of the fermentative acetate oxidizer *Thermacetogenium phaeum*. BMC Genomics.

[60] Westerholm M, Roos S, Schnürer A (2011). *Tepidanaerobacter acetatoxydans* sp. nov., an anaerobic, syntrophic acetate-oxidizing bacterium isolated from two ammonium-enriched mesophilic methanogenic processes. Syst Appl Microbiol.

[61] Müller B, Manzoor S, Niazi A, Bongcam-Rudloff E, Schnürer A (2015). Genome-guided analysis of physiological capacities of *Tepidanaerobacter acetatoxydans* provides insights into environmental adaptations and syntrophic acetate oxidation. PLoS One.

[62] Trischler R, Roth J, Sorbara MT, Schlegel X, Müller V (2022). A functional Wood-Ljungdahl pathway devoid of a formate dehydrogenase in the gut acetogens *Blautia wexlerae*, *Blautia luti* and beyond. Environ Microbiol.

[63] Liu X, Mao B, Gu J, Wu J, Cui S, Wang G (2021). *Blautia*-a new functional genus with potential probiotic properties?. Gut Microbes.

[64] Topçuoğlu BD, Meydan C, Nguyen TB, Lang SQ, Holden JF (2019). Growth kinetics, carbon isotope fractionation, and gene expression in the hyperthermophile *Methanocaldococcus jannaschii* during hydrogen-limited growth and interspecies hydrogen transfer. Appl Environ Microbiol.

[65] Holden JF, Sistu H (2023). Formate and hydrogen in hydrothermal vents and their use by extremely thermophilic methanogens and heterotrophs. Front Microbiol.

[66] Hensley SA, Jung JH, Park CS, Holden JF (2014). *Thermococcus paralvinellae* sp. nov. and *Thermococcus cleftensis* sp. nov. of hyperthermophilic heterotrophs from deep-sea hydrothermal vents. Int J Syst Evol Microbiol.

[67] Matthies D, Zhou W, Klyszejko AL, Anselmi C, Yildiz O, Brandt K (2014). High-resolution structure and mechanism of an F/V-hybrid rotor ring in a Na^+^-coupled ATP synthase. Nat Commun.

[68] Hess V, Gonzalez JM, Parthasarathy A, Buckel W, Müller V (2013). Caffeate respiration in the acetogenic bacterium *Acetobacterium woodii*: a coenzyme A loop saves energy for caffeate activation. Appl Environ Microbiol.

