# Supplementary material for: Reprogramming the metabolism of an acetogenic bacterium to homoformatogenesis
Source: ISME J. 2023 Apr 15;17(7):984–92. doi: 10.1038/s41396-023-01411-2 (PMC10284823; doi:10.1038/s41396-023-01411-2)
Supplement: Supplementary file 1 — Supplementary Information [file 41396_2023_1411_MOESM1_ESM.pdf]

Supplementary information for

Reprogramming the metabolism of an acetogenic bacterium to homoformatogenesis

Jimyung Moon, Anja Schubert, Lara M. Waschinger and Volker Müller[#]

Molecular Microbiology & Bioenergetics, Institute of Molecular Biosciences, Johann Wolfgang Goethe University, Max-von-Laue Str. 9, D-60438 Frankfurt, Germany

#Corresponding author. Mailing address: Department of Molecular Microbiology & Bioenergetics, Institute of Molecular Biosciences, Johann Wolfgang Goethe University, Max-von-Laue-Str. 9, D-60438 Frankfurt, Germany. Phone: 49-69-79829507. Fax: 49-69-79829306. E-mail: vmueller@bio.uni-frankfurt.de.

Running title: Homoformatogenesis in *A. woodii*

Table S1. Growth of the *A. woodii* wild type and the Δ*hdcr* mutant in carbonate-buffered complex medium with different substrates.

Substrates	Wild type	Δ*hdcr*
fructose (20 mM)	+	-
fructose (20 mM) + formate (50 mM)	+	+
H_2 + CO_2 (1 bar)	+	-
H_2 + CO_2 (1 bar) + formate (50 mM)	+	+
methanol (60 mM)	+	-
3,4,5-trimethoxybenzoic acid (5 mM)	+	-
3,4-dimethoxybenzoic acid (5 mM)	+	-
3,4,5-trimethoxycinnamic acid (5 mM)	+	-
1,2-dimethoxybenzole (5 mM)	+	-
vanillic acid (5 mM)	+	-
isovanillic acid (5 mM)	+	-
vanillin (5 mM)	+	-
ferulic acid (5 mM)	+	-
glycine betaine (50 mM)	+	+

Precultures were grown on 5 mM fructose + 10 mM formate.

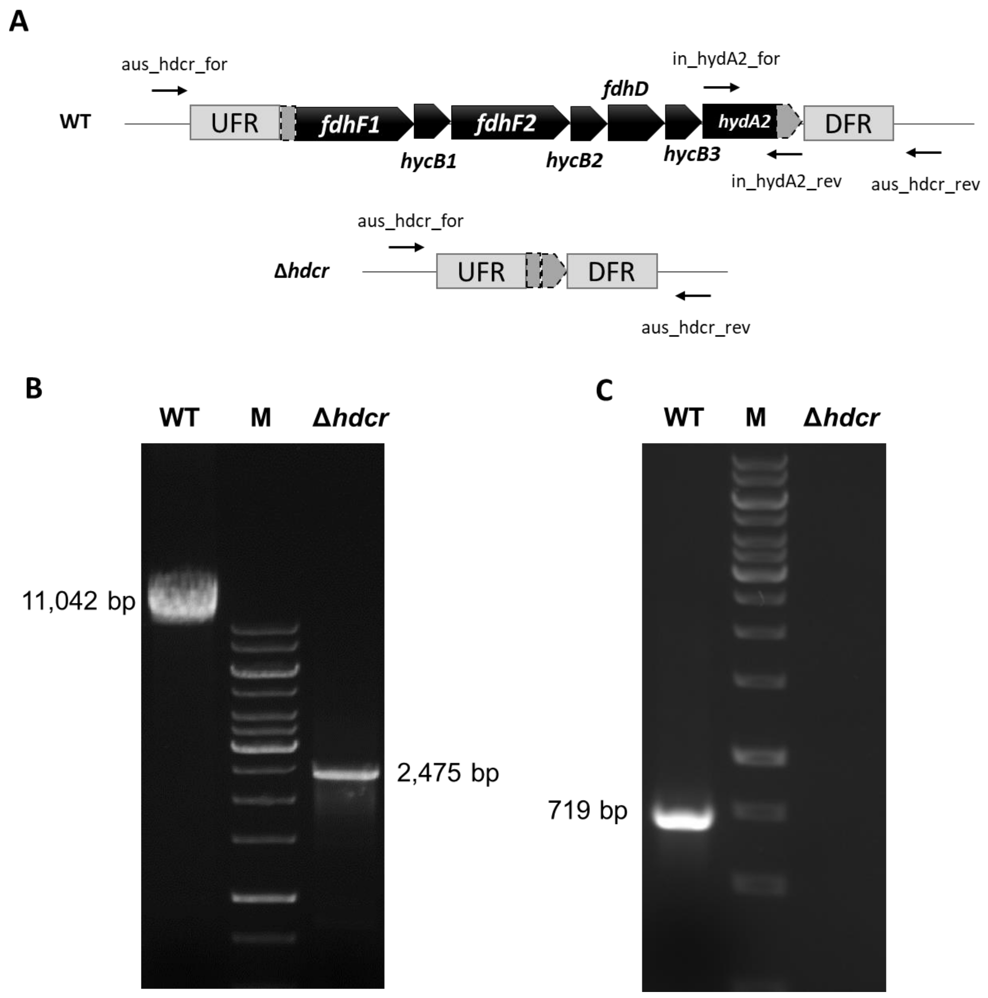

Figure S1. Deletion of the *hdcr* operon in the chromosome of *A. woodii*. (A) Genetic organization after deletion of the *hdcr* genes using plasmid pMTL_AW_KO_HDCR. After transformation, the integration of the plasmid into the chromosome was carried out by addition of 30 µg/µl thiamphenicol and complete deletion of all seven *hdcr* genes was forced by addition of 1 mg/ml 5-fluoroorotic acid (5-FOA). In the Δ*hdcr* mutant, only 3 bp of the *fdhF1* gene and 3 bp of the *hydA2* gene remained in the chromosome. Genotypic analyses of the Δ*hdcr* mutant were carried out by the PCR with primers binding outside the deleted region (B) (aus_hdcr_for and aus_hdcr_rev) or inside (C) (in_hydA2_for and in_hydA2_rev). Lane M shows the reference marker (GeneRuler 1-kb DNA ladder; Thermo Fisher Scientific, Waltham, MA, USA). WT, chromosomal DNA of the wildtype as template; Δ*hdcr*, chromosomal DNA of the Δ*hdcr* mutant as template.

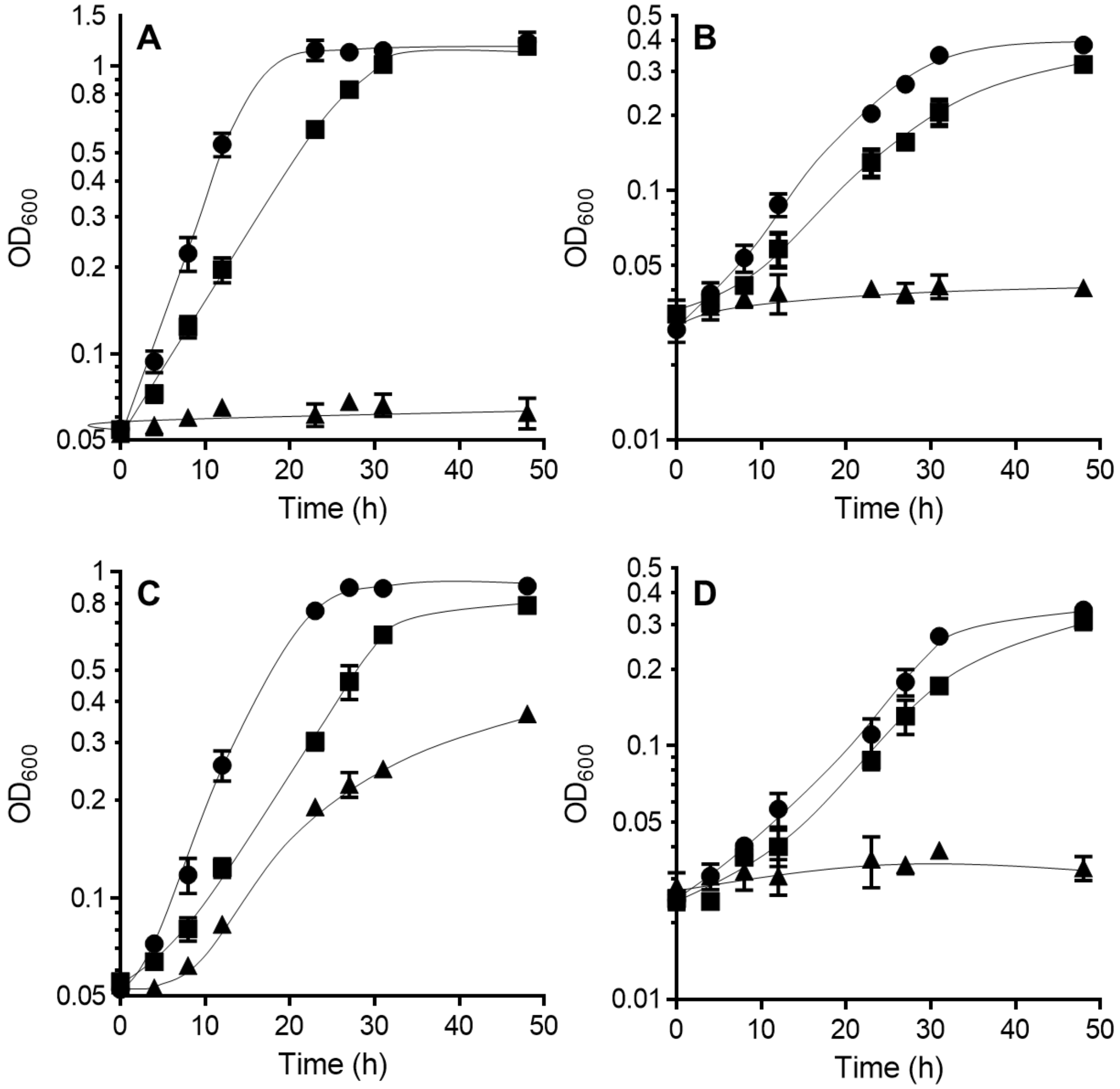

Figure S2. Phenotypic complementation of the Δ*hdcr* + pMTL84211_JM_Pptaack_hdcr strain. The wild type (●), the Δ*hdcr* mutant (▲), and the complementation strain Δ*hdcr* + pMTL84211_JM_Pptaack_hdcr (■) were grown in 5 ml complex medium in 16-ml Hungate tubes at 30 °C with (A) 20 mM fructose, (B) H_2+CO_2, (C) 50 mM glycine betaine, or (D) 100 mM formate as carbon and energy source. The growth experiments were performed in biological triplicates and a mean ± SD is presented.

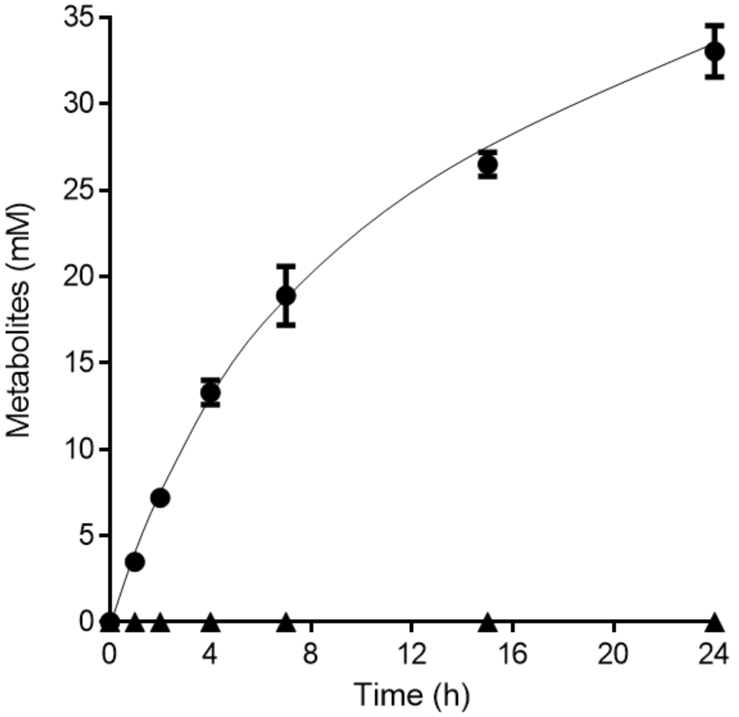

Figure S3. Conversion of glycine betaine in resting cells of *A. woodii* wild type. Cells of the wild type were grown in complex media with 50 mM glycine betaine and harvested in the early stationary growth phase. After washing, the cells were resuspended in 10 ml of cell suspension buffer in 120-ml serum flasks under a N_2/CO_2 atmosphere at a total protein concentration of 1 mg/ml. 50 mM glycine betaine was given to the cell suspensions as carbon and energy source. Acetate (●) and formate (▲) were determined at each time point. Each data point presents a mean ± SD; $n = 2$ independent biological replicates.

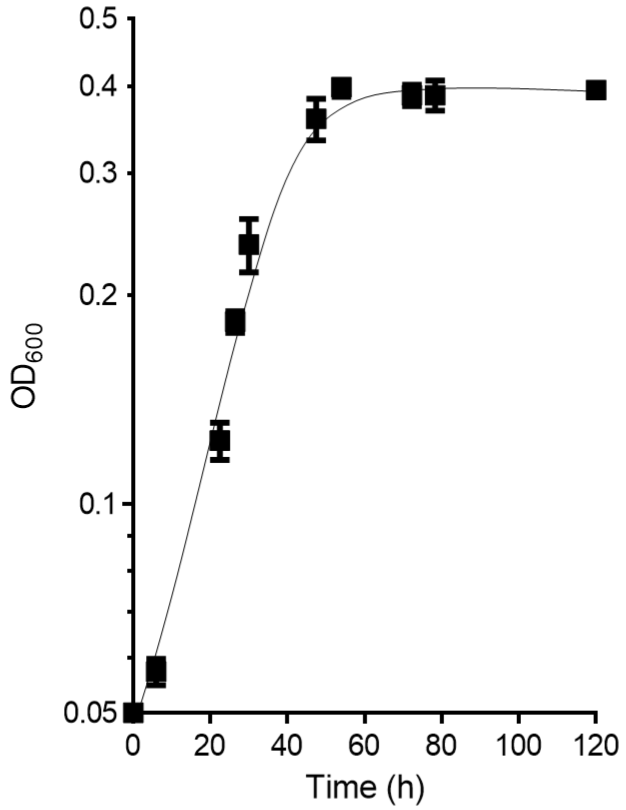

Figure S4. Growth of the Δ*hydBA*/*hdcr* mutant on glycine betaine. The Δ*hydBA*/*hdcr* mutant (■) was grown in 5 ml complex medium in 16-ml Hungate tubes at 30 °C with 50 mM glycine betaine as carbon and energy source. The growth experiments were performed in biological triplicates and a mean ± SD is presented.

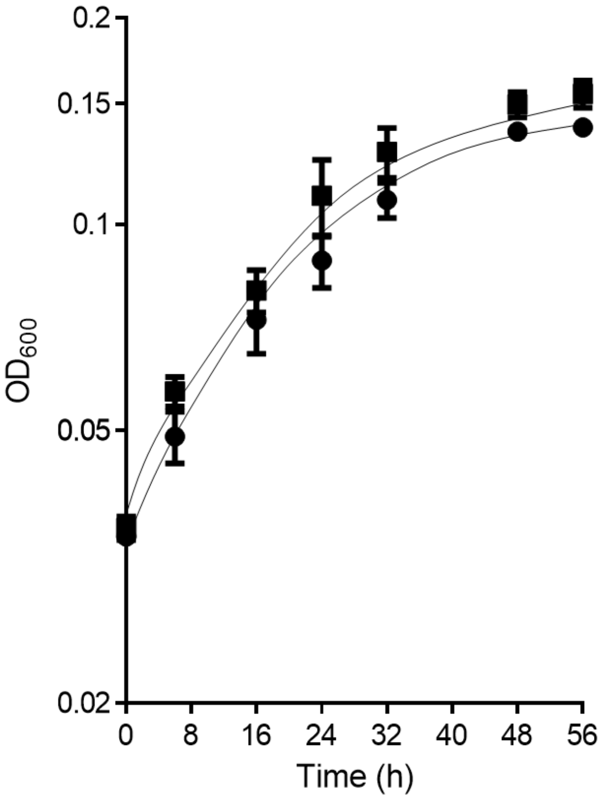

Figure S5. Growth of the Δ*hdcr* and Δ*hydBA*/*hdcr* mutants on glycine betaine in the presence of caffeate. The *hdcr* mutant (■) and the Δ*hydBA*/*hdcr* mutant (●) were grown in 5 ml complex medium in 16-ml Hungate tubes at 30 °C with 2 mM glycine betaine + 4 mM caffeate as carbon and energy source. The growth experiments were performed in biological duplicates and a mean ± SD is presented.

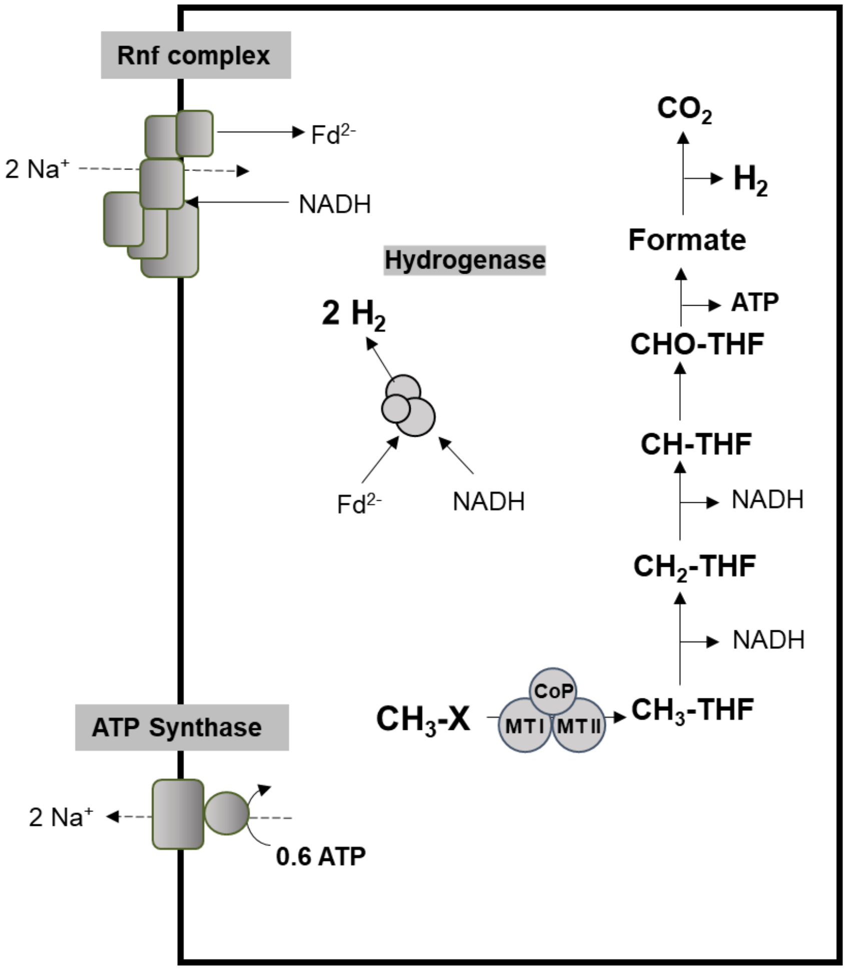

Figure S6. Biochemistry and bioenergetics of methyl group oxidation in *A. woodii*. CH_3-X, methyl groups; Fd, ferredoxin; THF, tetrahydrofolate; MTI, methyltransferase I; MTII, methyltransferase II; CoP, corrinoid protein. The stoichiometry of the ATP synthase is 3.3 Na^+/ATP (Matties *et al.*, 2014) and for the Rnf complex a stoichiometry of 2 Na^+/2e^- is assumed.
